# Association between mild action tremor and cognition in community dwelling older adults: results from the KORA-Age study

**DOI:** 10.1007/s00415-026-14056-w

**Published:** 2026-08-11

**Authors:** Gregor Kuhlenbäumer, Franziska Hopfner, Günther Deuschl, Karl-Heinz Ladwig, Birgit Linkohr, Annette Peters, Barbara Thorand, Marie-Theres Huemer

**Affiliations:** 1 Department of Neurology, Kiel University, Arnold-Heller Str. 3, 24114 Kiel, Germany; 2 Department of Neurology, LMU University Hospital, LMU Munich, Munich, Germany; 3 Department of Psychosomatic Medicine and Psychotherapy, Klinikum rechts der Isar, Technische Universität München, Munich, Germany; 4 German Center for Cardiovascular Research (DZHK), Partner site Munich Heart Alliance, Munich, Germany; 5 German Center for Diabetes Research (DZD), Munich-Neuherberg, Germany; 6 Institute of Epidemiology, Helmholtz Zentrum München, German Research Center for Environmental Health (GmbH), Ingolstädter Landstraße 1, 85764 Neuherberg, Germany; 7 Institute for Medical Information Processing, Biometry, and Epidemiology-IBE, Faculty of Medicine, LMU Munich, Munich, Germany; 8 German Center for Mental Health (DZPG), Partner site Munich-Augsburg, Munich, Germany

**Keywords:** Mild tremor, Subclinical, Archimedes spiral, Cognition

## Abstract

**Background:**

Essential tremor has been associated with cognitive impairment. Mild action tremor labelled physiological tremor is present in all humans. Tremor increases with age. It is unknown if mild action tremor is associated with cognitive performance in older people.

**Objective:**

To test the hypothesis that mild—mostly non-pathological—action tremor is associated with reduced cognitive performance in community-dwelling older adults.

**Methods:**

The Cooperative Health Research in the Region of Augsburg (KORA)-Age study assessed health-related parameters including cognition in individuals between 65 and 93 years of age. We measured tremor using semi-quantitative ratings of Archimedes-spiral drawings. Participants taking Parkinson medication, with a self-reported neurologic disease or with a spiral rating > 5 indicating certainly pathologic tremor were excluded. The modified Telephone Interview for Cognitive Status (TICS-m) was used to measure cognition. We analyzed the association between tremor and cognition using multivariable adjusted regression models in the whole population and after exclusion of participants with a spiral rating > 3. We employed stepwise backward variable selection to build a final regression model.

**Results:**

Tremor severity was associated with cognitive performance. Age, sex, Parkinson score, frailty, tremor score, physical activity, sleep-duration and previous stroke entered the final statistical model. Judged by the differences of adjusted r-squared between models, age was the most important predictor of cognition followed by sex, Parkinson score, frailty, and tremor severity. Sensitivity analyses excluding participants with likely pathological tremors did not attenuate the association between tremor severity and cognitive performance.

**Conclusion:**

Even mild action tremor severity is associated with reduced cognitive performance in older people. We hypothesize that aging-related brain changes are a common cause of cognitive impairment and tremor.

**Supplementary Information:**

The online version contains supplementary material available at https://doi.org/10.1007/s00415-026-14056-w.

## Introduction

Action tremor is a tremor occurring during posture or movement, but not at rest [1]. Mild, physiological hand tremor is an action tremor usually lacking clear visibility and persistence invariably present in humans, and increasing with age [1, 2]. Essential Tremor (ET) is a pathologic tremor characterized by a clearly visible and persistent symmetric action tremor of the arms, hands and other body parts [1]. In contrast, resting tremor which is not a subject of this study is a typical feature of Parkinson disease. However, patients with Parkinson disease often suffer from action tremor as well. A recent meta-analysis estimated the global prevalence of ET at 0.04% increasing to 2.87% at the age of 80 years and above [3]. However, the reported prevalences vary widely and an older meta-analysis reported a prevalence of 4.6% at an age above 65 years [4]. The most recent consensus statement of the movement disorders society defines ET as an etiologically heterogeneous syndrome [1]. In contrast other investigators view ET as a homogeneous neurological disease [5, 6]. Increasing evidence indicates that ET is associated with impairments of cognitive performance and possibly also with dementia [7–9]. ET patients showed significantly lower scores in the Mini-Mental State Examination [9] and a faster rate of cognitive decline [8] in a population-based study of older people in Spain. In a recent study in the USA ET patients also showed a higher cumulative prevalence of dementia and a higher annual conversion rate of mild cognitive impairment to dementia than controls [7]. This is in accordance with the hypothesis that ET is a neurodegenerative disease with evidence for neurodegenerative changes mainly affecting the cerebellum [10, 11]. The lateral hemispheres of the posterior cerebellar lobe are involved in cognition and lesions produce the cerebellar cognitive affective syndrome with deficits in motor planning, language processing, working memory, executive functions and emotional regulation [12, 13]. Typical pathological findings in ET patients are reduced Purkinje cell counts, pathological Purkinje cell morphology, so called “torpedos” and axonal alterations [6, 11]. However, a recent study reported pathological changes restricted to the motor areas of the cerebellum, but not present in its cognitive parts [10]. Other studies did not find neurodegeneration in ET at all [14, 15]. Archimedes-spiral drawings rated semi-quantitatively from 0 (no tremor) to 9 (maximal tremor) are a well-established and reliable instrument to assess action tremor [16]. We have recently shown that tremor strength measured via the Archimedes Spiral Rating (ASR) is a continuum and that the upper limit of the 95% confidence interval—hence the 97.5 percentile—of spiral scores in individuals above 60 years of age unselected for tremor and without known tremor-causing diseases or drugs lies between ASRs of 4 and 5 [2]. We investigated whether the severity of mild action tremor is associated with cognitive performance measured in participants of the population-based KORA-Age study who were unselected for tremor. Participants with known tremor-causing conditions were excluded to focus on non-pathological action tremor. A sensitivity analysis was then restricted to participants with an ASR ≤ 3, a threshold corresponding approximately to the population median in this age group, thereby limiting the analysis to individuals with very mild action tremor.

## Methods

### Study population

The data were derived from 1079 individuals who participated in the first examination of the KORA-Age study, a population-based cohort study of health and aging in older people 65–93 years of age in the city of Augsburg and two adjacent counties in southern Germany [17]. The study population was a sex- and age-stratified randomly selected group of participants born in 1943 or earlier who participated in any of the four preceding Monitoring of Trends and Determinants in Cardiovascular Disease (MONICA)/KORA Augsburg studies (MONICA S1 (1984/85), MONICA S2 (1989/90), MONICA S3 (1994–1995) or the KORA S4 study (1999–2001)) [17]. In the KORA-Age examination, the participants drew an Archimedes spiral to assess the tremor. A postal health questionnaire and a telephone interview were conducted to determine somatic multimorbidity and mental health status of the participants. All variables used in this study were assessed at the same time in 2008 at study inclusion.

### Cognitive Performance (Outcome Variable)

The TICS-m in German is a direct translation of the English TICS-m [18]. The TICS-m is a modified version of the TICS [19], which includes two additional tasks: immediate and delayed verbal recall. The TICS-m contains the following items: (i) name, date, age and phone number (9 points); (ii) counting backward (2 points); (iii) first, a 10-word list learning exercise and then a delayed recall of that word list (20 points); (iv) serial sevens (5 points); (v) responsive naming (4 points); (vi) repetition (2 points); (vii) current German President and Chancellor (4 points); (viii) finger tapping (2 points) and (ix) word opposites (2 points).The TICS-m includes four domains: orientation; memory (registration, recent memory, and delayed recall); attention/calculation; and language (semantic memory, comprehension, and repetition) [20]. We used the TICS-m assessed via telephone interview adjusted for education as a continuous variable with a range of 0–50 points as described in detail by Lacruz et al. [18].

### Tremor

An Archimedes spiral drawn with the preferred hand on paper was rated from 0 to 9 by one of the authors (FH) who is very experienced in spiral rating [2, 21, 22]. We have previously shown good interrater and intrarater concordance of ASR and determined the age and sex adjusted approximate 95% confidence intervals of spiral ratings in 1343 participants of a population based study aged 18 to > 80 years [2, 21]. In KORA-Age, the participants were instructed to start the drawing of the spiral at the marked point in the middle, and then draw the line between the pre-sketched lines until the point located outside. The line should be drawn swiftly without crossing over the pre-sketched lines. In addition, the participants were instructed to draw consistently without putting down the pen, while it was allowed to hold the paper with the other hand. Pictures with examples of spiral drawings are shown in the supporting information of Hopfner et al. 2024 [2].

### Covariables

(i) Age in years. (ii) Sex (binary, reference level female). (iii) Physical activity was assessed as a continuous variable using the Physical Activity Score in the Elderly (PASE) [23, 24]. Morbidity related variables: (iv) Frailty was determined using an adapted version of the frailty phenotype [25] using the categories “non-frail”, “prefrail”, and “frail”, as described by Vogt et al. [26]. The “frail” group was small (*n* = 24) and the difference in TICS-m between the “prefrail” and “frail” group was minor (mean delta TICS-m = 0.7 points) and not significant (*p* = 0.755). Therefore, we categorized the frailty variable into two groups: “non-frail” and “prefrail/frail”. (v) For multimorbidity we used a continuous score developed by Kirchberger et al. [27] without “mental disease” and, therefore, ranging from 0 to 12 points. (vi) Weighted Parkinson score assessing motor function as published by Höglinger et al. was included as a continuous variable ranging from 0 to 67 points [28]. (vii) Self-reported previous stroke was recorded as a binary variable. (viii) Hypertension was assessed as a binary variable defined as actual hypertension (> = 140/90 mm Hg) or medically treated known hypertension according to ISH-WHO1999 [29]. (ix) Body-Mass-Index (BMI) as kg/m^2^. Mental health related variables: (x) Depression was assessed with the German short form of the Geriatric Depression Scale (GDS) as a continuous variable ranging from 0 to 15 points [30]. (xi) Loneliness was assessed using the German version of the UCLA loneliness scale as a binary variable with > 21 points indicating loneliness[31]. Drugs and substance abuse related variables: (xii-xiv) ß-Blocker, thyroid hormone and thyreostatics medication were recorded as binary variables. (xv) Alcohol consumption was assessed quantitatively following the method published by Doring et al. [32] and subsequently divided into three categories as follows: 0 = abstinence, 1 = less than 20 g/day for females and less than 40 g/day for males, 2 = greater alcohol consumption than category 1.

Sleep-related variables were assessed using the Upsala Sleep Inventory [33] as described by Johar et al. for KORA-Age [34]. (xvi) Daily hours of sleep were recorded as continuous variables. (xvii) Problems falling asleep was used as a categorical variable with the levels: 1 = almost never, 2 = sometimes, 3 = often. (xviii) Problems sleeping through the night had the same levels as problems falling asleep. Since the difference in TICS-m between “almost never” and “sometimes” for problems sleeping through the night was very small (mean delta TICS-m = 0.4 points) and not significant (*p* = 0.58), we merged the levels to “almost never” versus “sometimes/often”.

### Statistical analysis

All analyses were performed in RStudio 2023.03.01. Variable distribution was assessed using visual histogram analysis. We visualized the relationship between all categorical predictors and the continuous outcome TICS-m with boxplots overlaid with scatterplots and with histograms (data not shown). The relationship with all continuous predictors was visualized using scatter plots with regression lines and locally estimated scatterplot smoothing (LOESS, not shown) to assess linearity. Basic statistical tests (*t* test, Fisher’s exact test, simple linear regression) are specified in the text together with coefficients and *p* values. The dependent variable (TICS-m) was normally distributed and we used linear regression/ANOVA with Tukey’s post-hoc test to assess group differences for categorical predictors and linear regression for continuous predictors. We used standard diagnostic plots (fitted values versus residuals and standardized residuals, quantile–quantile and leverage versus standardized residuals) for regression diagnostics and the studentized Breusch-Pagan test (BPT) to assess heteroscedasticity. After identifying all single variables that were significantly associated with TICS-m, we removed all participants with missing values in at least one of these variables and repeated the univariate analyses. The results of this single-factor analysis after exclusions are shown in the results section (Table 1). Variables significantly associated with TICS-m entered a multiple regression model without any missing values. We used a manual backward selection procedure with a *p* value cut-off of ≤ 0.05 to determine the final model. Interaction between ASR and beta-blockers was assessed by adding an interaction term to the regression. Estimated marginal means were calculated using the emmeans R package. We performed the following sensitivity analyses: (i) excluding individuals with a previous self-reported stroke (*n* = 63), (ii) excluding participants with a weighted Parkinson score > 21 (*n* = 180) because this value provided an optimal cut-off (Sensitivity 91.7%, Specificity: 85.9%) for Parkinson’s disease in a previous German study [28], (3) excluding participants with an ASR of > 3 (*n* = 13) because this rating was previously identified as the lower limit of a potentially pathogenic tremor in a large study [2]. We present the results of regression models in the results section in the format: “beta (lower bound of 95% confidence interval, upper bound of 95% confidence interval), per variable name/unit, *p* value” with or without *p* values depending on context.

**Table 1:** Variables analyzed in this study: descriptive statistics and association with TICS-m

Parameter	Descriptive statistic	Mean ± SD TICS-m per group	*β* (95% CI)	Adj. *R*^2^	*p* value
Cognitive performance (TICS-m)	35.5 ± 5.0	NA	NA	NA	NA
Age (years)	75 ± 6	NA	− 0.26 (− 0.31; − 0.22)	0.113	< 0.001
Sex			− 2.00 (− 2.64; − 1.36)	0.039	< 0.001
Female	439 (49.1%)	36.5 ± 5.0			
Male	455 (50.9%)	34.5 ± 4.8			
Parkinson score (per point)	11 ± 12	NA	− 0.10 (− 0.13; − 0.08)	0.062	< 0.001
Frailty			− 2.65 (− 3.29; − 2.01)	0.067	< 0.001
Non-frail	539 (60.3%)	36.5 ± 4.8			
Prefrail/frail	355 (39.7%)	33.9 ± 4.8			
Tremor (per ASR, linear)	2.2 ± 0.8	NA	− 1.37 (− 1.76; − 0.97)	0.050	< 0.001
Daily hours of sleep (per hour)	7.7 ± 1.4	NA	2.31 (0.79; 3.83) (lin.) − 0.16 (− 0.26; − 0.06) (quad.)	0.013	0.001
Physical activity (per point PASE)	121 ± 55	NA	0.008 (0.002; 0.014)	0.006	0.009
Previous stroke			− 2.71 (− 3.97; − 1.44)	0.018	< 0.001
No stroke	831 (93.0%)	35.6 ± 4.9			
Stroke	63 (7.0%)	32.9 ± 4.8			
Multimorbidity (per point Nr. of diseases, 0–12)	2 ± 1	NA	− 0.60 (− 0.83; − 0.36)	0.025	< 0.001
Depression (per point GDS)	2.18 ± 2.26	NA	− 0.35 (− 0.49; − 0.21)	0.024	< 0.001
Thyroid hormone use			1.40 (0.59; 2.21)	0.012	< 0.001
No thyroid hormone use	714 (79.9%)	35.2 ± 4.9			
Thyroid hormone use	180 (20.1%)	36.6 ± 5.0			
Beta-blocker use			− 1.02 (− 1.68; − 0.35)	0.009	0.003
No beta-blocker use	555 (62.1%)	35.8 ± 5.0			
Beta-blocker use	339 (37.9%)	34.8 ± 4.9			
Problems sleeping through			− 0.96 (− 1.61; − 0.31)	0.008	0.010
Almost never/sometimes	493 (55.1%)	35.8 ± 4.8			
Often	401 (44.9%)	34.9 ± 5.1			
Loneliness (UCLA, dichotomized)			− 1.05 (− 2.52; − 0.23)	0.006	0.012
Not lonely	720 (80.5%)	35.7 ± 4.9			
Lonely	174 (19.5%)	34.6 ± 5.0			
Alcohol consumption			NA*1	0.002	0.158
Abstinent	313 (35.0%)	35.3 ± 5.1			
Low	459 (51.3%)	34.4 ± 5.0			
Moderate/high	122 (13.6%)	36.3 ± 4.7			
Body-mass-index (kg/m^2^)	28.41 ± 4.27	NA	− 0.05 (− 0.12; 0.03)	0.001	0.222
Problems falling asleep			NA*1	0.001	0.236
Almost never	436 (48.8%)	35.5 ± 4.9			
Sometimes	304 (34.0%)	35.7 ± 4.9			
Often	154 (17.2%)	34.9 ± 5.2			
Hypertension			0.04 (− 0.070; 0.78)	− 0.001	0.913
Normotension	230 (25.7%)	35.43 ± 5.0			
Hypertension	664 (74.3%)	35.47 ± 4.9			
Thyreostatics use			0.14 (− 2.96; 3.24)	− 0.001	0.928
No thyreostatics use	884 (98.9%)	35.5 ± 5.0			
Thyreostatics use	10 (1.1%)	35.6 ± 4.7			

Descriptive statistics are shown as mean ± standard deviation for continuous variables and as N (%) for categorical variables. Linear regression was applied for continuous variables, and ANOVA for categorical variables with the dependent variable TICS-m. Variables are ordered by increasing *p* value

*1—no beta because the factors have three levels and a post-hoc analysis was not performed since the ANOVA *p* value was not significant

## Results

**Fig. 1:**
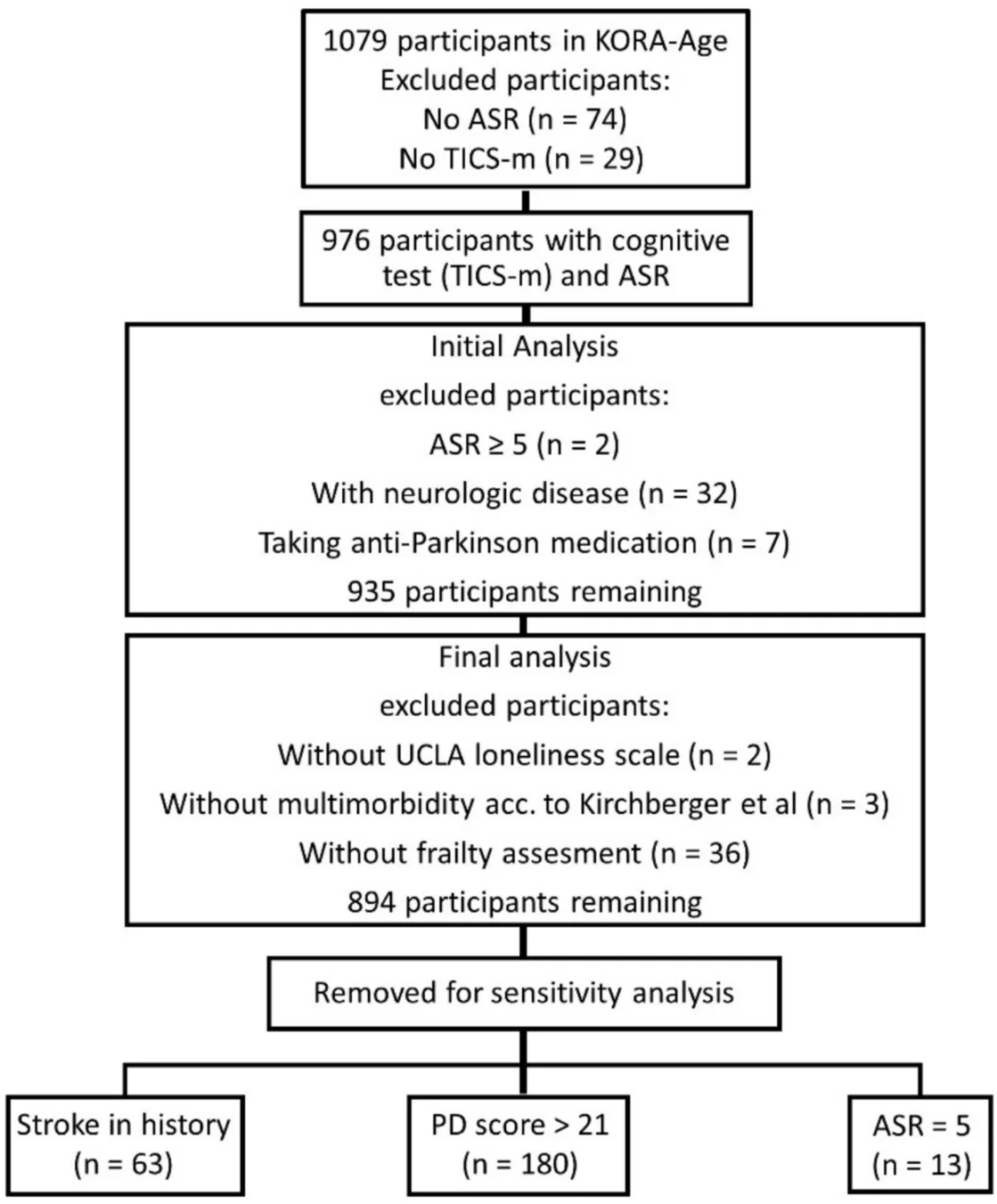
Flowchart of the participant selection process. Initial simple regression analyses are found in Table 1. Final analysis: number of participants that entered the final simple- and multivariable analyses. The last row indicates the number of individuals per phenotype removed in the sensitivity analyses. *ASR* Archimedes Spiral Rating, PD: Parkinson’s disease, *TICS-m* modified Telephone Interview for Cognitive Status

**Fig. 2:**
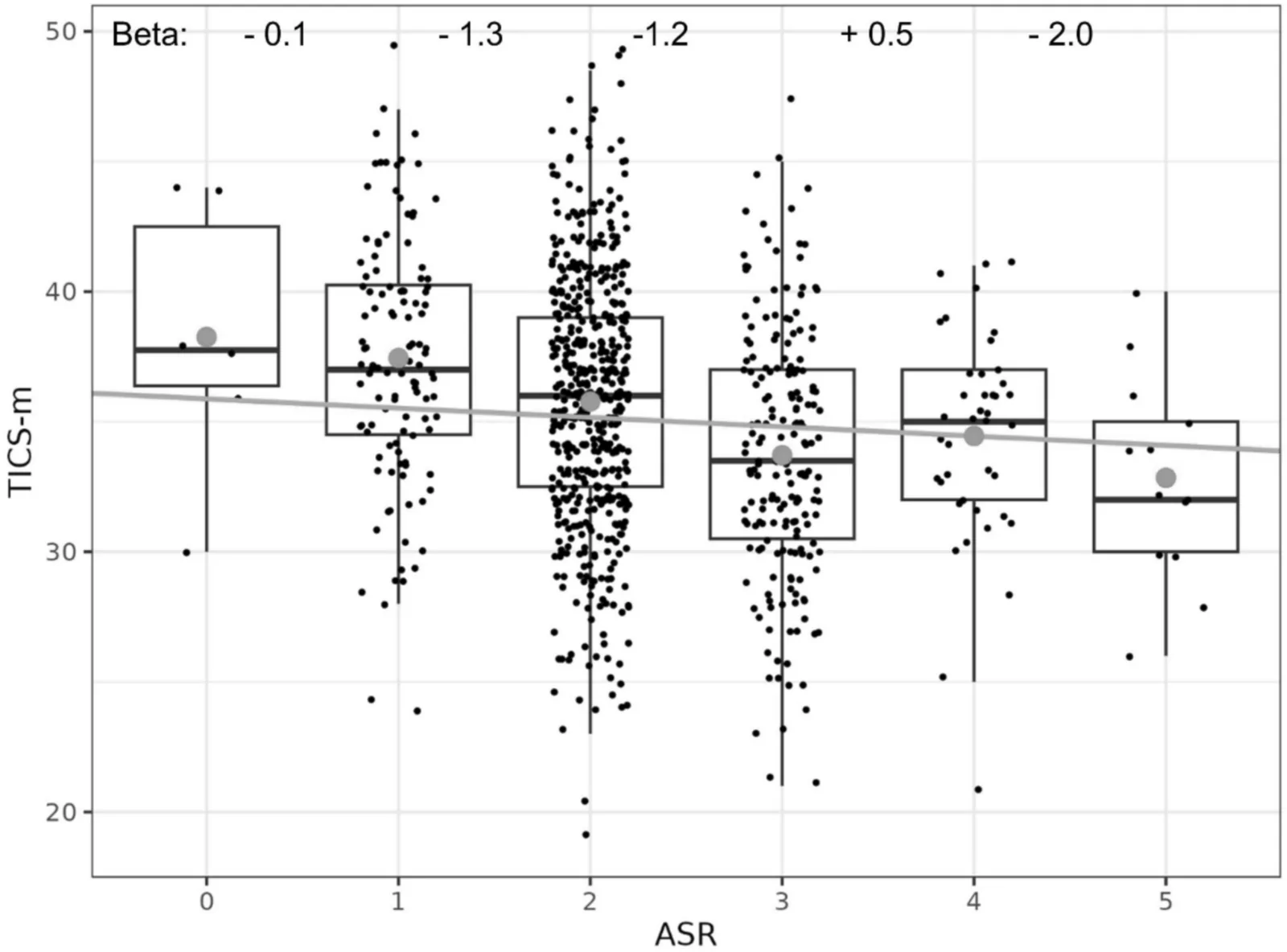
Unadjusted relationship between ASR and TICS-m. ASR viewed as a categorical variable (boxplots) and as the mean (grey dots) of a continuous variable (scatterplots and linear regression line). Grey dots: mean of TICS-m per ASR category. Beta’s are for differences between ASR groups, e.g., − 0.1 for ASR 1 vs 0

**Fig. 3:**
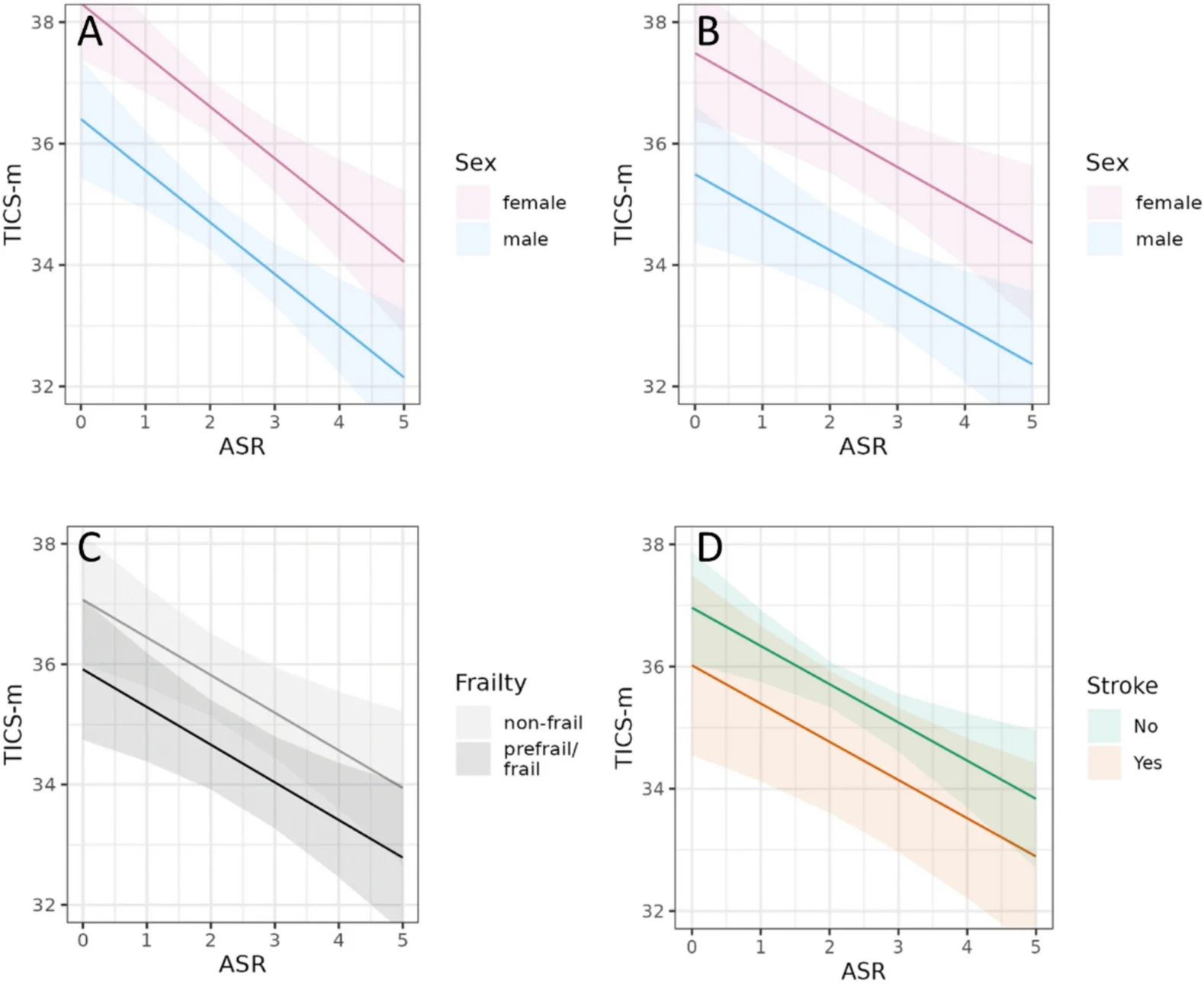
Relationship of ASR and TICS-m in the estimated marginal means analysis. **A:** ASR per TICS-m for females and males adjusted for the mean of age only; **B**–**D**: adjusted for the means of age, physical activity, daily hours of sleep, and the median of the Parkinson score and averaged over the levels of the categorical covariates not displayed in the figures. **B:** for females and males; **C:** for non-frail vs. prefrail/frail participants;** D**: for participants without and with stroke in the history

We analyzed whether mild action tremor in older people unselected for tremor in the KORA-Age study measured using semi-quantitative rating of Archimedes spiral drawings (ASR, 0 to 5) is associated with cognitive status measured using the TICS-m. Figure 1 shows a flowchart of the analyses with inclusion and exclusion criteria. Supplementary Fig. 1 shows a co-occurrence matrix of missing values demonstrating a non-random missingness pattern. Supplementary Table 1 compares variables between included and excluded participants. While the comparison is flawed by the co-occurrence of missing values it still demonstrates e.g. that excluded participants are older, show more tremor, lower TICS-m, a higher Parkinson score, a lower physical activity and a higher percentage of individuals with a previous stroke. In summary, excluded participants are less healthy than included participants. The basic characteristics and the results of the simple regression analyses including 935 participants for TICS-m as the dependent variable and all variables potentially influencing cognition or tremor from the KORA-Age study as independent variables are shown in Table 1. Figure 2 shows the unadjusted relationship between ASR and TICS-m scores. First, we considered a basic model including only age, sex and tremor as predictors of TICS-m. Increasing age (− 0.24 (− 0.29; − 0.20) TICS-m per year, *p* < 0.001), male sex (− 1.90 (− 2.50; − 1.31) TICS-m, *p* < 0.001), and higher ASR (− 0.85 (− 1.23; -0.47) TICS-m per point increase, *p* < 0.001) were all significantly associated with a lower TICS-m. Next, we performed a manual backward selection with an exclusion threshold of *p* < 0.05 of a model with all variables significantly associated with TICS-m (Table 1, *p* < 0.05) and added the variables daily hours of sleep, and physical activity manually to the model because they were previously reported to be associated with cognition [34–36] and yielded *p* values of < 0.1 in the present analysis. A quadratic term was required for daily hours of sleep because our data and previous studies suggest that both, too much and too little sleep were associated with lower cognitive performance [36]. Previous stroke was added as well because it is a known predictor of cognition [37, 38], and was associated with a fairly strong reduction of the TICS-m by − 0.942 in our study. The final model (Table 2A) including 894 participants contained the following predictors: age (− 0.19 (− 0.24; − 0.14), TICS-m per year, *p* < 0.001), male sex (− 1.99 (− 2.59; − 1.40), TICS-m, *p* < 0.001), Parkinson-score (− 0.05 (− 0.08; − 0.02) TICS-m per point, *p* < 0.001), frailty (− 1.16 (− 1.84; − 0.48), TICS-m for “prefrail/frail” versus “non-frail”, *p* < 0.001), tremor (ASR, − 0.63 (− 1.01; − 0.25), TICS-m per point, *p* = 0.001), daily hours of sleep with a quadratic (− 0.08 (− 0.17; 0.01), TICS-m per hour, *p* = 0.087) and a linear term (1.18 (− 0.20, 2.56), TICS-m per hour, *p* = 0.094), physical activity (− 0.01 (− 0.01; 0.00), TICS-m per point, *p* = 0.074) and history of stroke (− 0.94 (− 2.12; 0.23), TICS-m, *p* = 0.116). Supplementary Fig. 2 displays the reduction in adjusted *R*^2^ and the increase of the root mean squared error during the backward-selection procedure. Figure 3 shows the results of the estimated marginal means analyses predicting the effect of ASR on TICS-m adjusted for typical values of the covariates. Next, we conducted three sensitivity analyses to evaluate the robustness of the association between ASR and cognitive performance by excluding individuals based on covariates. First, we excluded participants with a history of stroke (*n* = 63, Table 2B) which did not lead to a large change of the coefficient. The coefficient of the ASR was slightly reduced from − 0.63 (− 1.01; − 0.25) TICS-m with inclusion of participants with a history of stroke to − 0.59 (− 0.99; − 0.19) without these individuals. Second, we removed all participants with a Parkinson score > 21 (*n* = 180, Table 2C) [28] which led to a larger negative coefficient of − 0.09 (− 0.14; − 0.03) TICS-m per point for the Parkinson score compared to the whole sample of − 0.05 (− 0.08; − 0.02) TICS-m, while the effect of the ASR was similar with − 0.70 (− 1.15; − 0.25) TICS-m per point ASR. Finally, we removed all participants with an ASR > 3 (Table 2D) to get a subsample with very mild action tremor. Coefficients in this subsample did again not differ much from the ones in the whole sample. The absolute coefficient of the ASR (− 1.08 (− 1.58; − 0.58) TICS-m per point ASR) was numerically larger compared to the whole sample (− 0.63 (− 1.01; − 0.25) TICS-m per point ASR). Beta-blockers are known tremor suppressants and associated with TICS-m in the unadjusted analysis (Table 1). In our sample beta-blockers were not associated with tremor after adjustment for age and sex (0.07 (− 0.04; 0.18), *p* = 0.19) Table 3A). Adding beta-blockers to the final model shown in Table 2A did not change the association between ASR and TICS-m (− 0.62 (− 1.00; − 0.24) TICS-m per ASR point, *p* = 0.001, Table 3B) compared to the final model (− 0.63 (− 1.01; − 0.25) TICS-m per ASR point, *p* = 0.001, Table 2A) and beta-blockers were not significantly associated with TICS-m (− 0.36 (− 0.97; 0.25), *p* = 0.25, Table 3B). Adding an interaction term between ASR and beta-blockers did not lead to significant main effects of beta-blockers (− 1.35 (− 3.14; 0.45), *p* = 0.14, Table 3C), nor a significant interaction term (0.44 (− 0.31; 1.20), *p* = 0.25, Table 3C). Beta-blockers were also not associated with TICS-m in the final model with the addition of beta-blockers after removing ASR (− 0.37 (− 0.99; 0.24), *p* = 0.24, Table 3D). Thyroid hormone use might cause tremor. In our sample thyroid hormone use was neither associated with tremor (-0.05 (− 0.69; 0.49) per ASR point, *p* = 0.489) nor with TICS-m (Table 1). The variance inflation factor (VIF, GVIF for models with interaction terms) was < 2 for all variables in multiple variable analyses indicating lack of multicollinearity. The scale-location plots (predicted values versus square-root of standardized residuals) as well as the *p* values of all Breusch–Pagan tests > 0.05 indicated no significant heteroscedasticity (not shown). The residuals versus leverage plots indicated no outliers in the sample space (not shown).

**Table 2:** Results of the multiple regression analyses in the whole sample and the sensitivity analyses

	A: Whole study population		B: Without participants with a history of stroke		C: Without participants with Parkinson-score > 21		D: Without participants with ASR > 3	
	Adjusted *R*^2^: 0.21		Adjusted R2: 0.20		Adjusted R2: 0.18		Adjusted R2: 0.22	
	*β* (95% CI)	*p* value	*β* (95% CI)	*p* value	*β* (95% CI)	*p* value	*β* (95% CI)	*p* value
Age (per year)	− 0.19 (− 0.24; − 0.14)	< 0.001	− 0.20 (− 0.25; − 0.15)	< 0.001	− 0.19 (− 0.25; − 0.14)	< 0.001	− 0.20 (− 0.25; − 0.15)	< 0.001
Sex (male vs. female)	− 1.99 (− 2.59; − 1.40)	< 0.001	− 2.02 (− 2.64; − 1.41	< 0.001	− 1.85 (− 2.51; − 1.19)	< 0.001	− 1.99 (− 2.60; − 1.38)	< 0.001
Parkinson Score (per point)	− 0.05 (− 0.08; − 0.02)	< 0.001	− 0.04 (− 0.07; − 0.02)	0.003	− 0.09 (− 0.14; − 0.03)	0.002	− 0.06 (− 0.08; − 0.03)	< 0.001
Frailty (frail/prefrail vs. non-frail)	− 1.16 (− 1.84; − 0.48)	< 0.001	− 1.24 (− 1.94; − 0.53)	0.001	− 1.15 (− 1.91; − 0.39)	0.003	− 1.09 (− 1.80; − 0.39)	0.002
Tremor (per ASR)	− 0.63 (− 1.01; − 0.25)	0.001	− 0.59 (− 0.99; − 0.19)	0.004	− 0.70 (− 1.15; − 0.25)	0.002	− 1.08 (− 1.58; − 0.58)	< 0.001
Sleep (hours, linear term)	1.18 (− 0.20, 2.56)	0.094	0.69 (− 0.87; 2.25)	0.384	1.44 (− 0.18, 3.06)	0.081	1.43 (− 0.01; 2.86)	0.051
Sleep (hours, quadratic term)	− 0.08 (− 0.17; 0.01)	0.087	− 0.05 (− 0.15; 0.05)	0.359	− 0.09 (− 0.20; 0.01)	0.080	− 0.09 (− 0.18; 0.00)	0.054
Physical activity (per point)	− 0.01 (− 0.01; 0.00)	0.074	− 0.01 (− 0.01; 0.00)	0.060	− 0.01 (− 0.02; 0.00)	0.008	− 0.01 (− 0.01; 0.00)	0.033
Stroke (yes vs. no)	− 0.94 (− 2.12; 0.23)	0.116	NA	NA	− 0.72 (− 2.27; 0.83)	0.361	− 0.91 (− 2.18; 0.36)	0.159

Variables are ordered by increasing *p* value in the whole study population

**Table 3:** Influence of beta-blockers on the association between ASR and TICS-m

Additional models	ASR		Beta-blockers		Interaction	
	*β* (95% CI)	*p* value	*β* (95% CI)	*p* value	*β* (95% CI)	*p* value
A: ASR ~ age + sex + beta-blockers	NA	NA	0.07 (− 0.04; 0.18)	0.191	NA	NA
B: Final model + beta-blockers	− 0.62 **(**− 1.00; − 0.24**)**	0.0014	− 0.36 (− 0.97; 0.25)	0.252	NA	NA
C: Final model + beta-blockers + interaction	− 0.78 **(**− 1.25; − 0.31**)**	0.0012	− 1.35 **(**− 3.14; 0.45**)**	0.142	0.44 (− 0.31; 1.20)	0.251
D: Final model + beta-blockers − ASR	NA	NA	− 0.37 **(**− 0.99; 0.24**)**	0.235	NA	NA

## Discussion

This study observed that mild action tremor is independently associated with lower cognitive performance in older people unselected for tremor from the population-based KORA-Age study. This holds true not only for mild to moderate tremors (ASR 0 to 5) but also for very mild tremors (ASR 0 to 3) [2]. Age, sex, and frailty are well-known predictors of cognition [39–43]. Overt Parkinson’s disease has been associated with tremor and cognitive decline [44, 45]. Therefore, participants taking medication for Parkinson’s disease were excluded prior to all analyses. However, previous studies showed that subtle signs of extrapyramidal dysfunction, such as gait and posture impairment, mild rigidity, and reduced leg pendulousness, are also predictors of cognition [46, 47]. In our study, tremor was equally strongly associated with lower cognitive performance after removal of participants with high scores on the Parkinson scale, which suggests that the effect of tremor on TICS-m is largely independent of extrapyramidal motor function assessed by the Parkinson scale [28]. Stroke is a well-known factor associated with cognition [38]. Since stroke can also cause tremor, we performed a sensitivity analysis without participants with prior stroke which did not influence the association of tremor with cognition, highlighting the independent role of tremor. In contrast to other studies, we did not observe the closely connected factors of depression or loneliness to be associated with cognition in the final model [48–50]. This might be due to the relatively low scores of the study population on the Geriatric Depression Scale with a mean of 2.2 out of 15 points. Analysis of beta-blockers and knowledge of their well-known pharmacological effects shows that beta blockers are a marker for reduced cognitive performance, but that the reduction in cognitive performance is caused by the older age and poorer health of the study participants receiving beta-blockers [51]. In our study beta-blockers were associated with TICS-m in the unadjusted analysis, but our detailed analyses of beta-blockers in adjusted models do not support a significant modification of the association between ASR and TICS-m by beta-blockers in our study. The same applies to thyroid hormones which were not associated with TICS-m nor with the ASR in any analysis. Physiologic tremor is caused by cardioballistic forces and/or physiologic oscillatory brain activity. Pathologic tremor can be a consequence of neurologic disease, causing rhythmic brain activity coherent with the electromyogram of the muscles moving the affected limb. The most common conditions causing tremor are ET and PD. Some pathologic studies indicated mild signs of neurodegeneration in ET patients in the cerebellum while others do not, while PD as the classical neurodegenerative disease is associated with cognitive decline and inevitably associated with impaired cognition if the patients live long enough [52] A number of previous studies found an association between ET and cognitive impairment [9, 53–55]. While older pathologic studies of ET do not differentiate between regions of the cerebellum, the most recent one explicitly emphasizes that pathology affects regions of the cerebellum associated with motor function, but not the ones implied in cognition. Therefore, it is currently not clear if cerebellar pathology found in ET is able to explain cognitive deficits. In contrast to previous studies of ET, we analyzed mild action tremor characterized by an ASR between 1 and 5 or in the sensitivity analysis an ASR of 1 to 3. While an Archimedes spiral alone is not sufficient for the diagnosis of tremor entities (e.g. ET) it is a good measure of tremor severity. We have previously shown in a large study using the same spiral rater (author FH), that the mean ASR in older individuals is 2.45 ± 0.73 (age 60–69, mean, standard deviation) to 2.91 ± 0.86 (age ≥ 80 years, mean, standard deviation). Therefore, the ASR in most individuals in the current study is certainly within the limits of one standard deviation of their age group defined in our previous work [2] and an upper limit of the ASR of 3 in our sensitivity analysis limits the range to mild action tremors. The cause of the association between mild tremor and reduced cognition remains unknown. A hypothetical cause are aging-associated changes of brain circuits causing tremor and impaired cognition.

This study has a number of strengths and limitations. Major strengths are the well-established, population-based study sample with a large amount of available covariates assessed using established instruments and few missing data. The main weaknesses are the missing longitudinal data of the ASR and the problem of reliable tremor strength measurement. However, the ASR is a proven, validated and commonly used instrument to assess tremor severity [2, 16]. In addition, one could name the exclusion of participants with missing data for key variables, e.g. ASR and TICS-m. Excluded participants are less healthy than the included ones and we think that it is likely that they would exhibit more tremor and worse cognitive performance further strengthening the association between tremor and cognition. However, we are unable to test this hypothesis.

In summary, we show that even mild non-pathological tremor is associated with lower cognitive performance in older people above age 65 in the KORA-Age study. One of the possible interpretations is that aging-associated brain changes are the common cause for tremor and cognitive decline. Future longitudinal studies should assess the causal relationship between age, mild tremor and cognition. We conclude that the severity of mild action tremor within the range of the mean plus/minus one standard deviation of the ASR in this age group is associated with decreased cognitive performance assessed using the TICS-m. We recognize that we cannot differentiate between pathological tremors classified according to the criteria of the Movement Disorders Society [1] and physiological tremor.

## Supplementary Information

Below is the link to the electronic supplementary material.

Supplementary file1 (DOCX 234 KB)

## Data Availability

The informed consent given by KORA study participants does not cover data posting in public databases. However, data are available upon request from KORA.PASST (https://helmholtz-muenchen.managed-otrs.com/external/) by means of a project agreement. Requests should be sent to kora.passt@helmholtz-munich.de and are subject to approval by the KORA Board.
