# Supplementary material for: Association between mild action tremor and cognition in community dwelling older adults: results from the KORA-Age study
Source: J Neurol. 2026 Aug 11;273(9):523. doi: 10.1007/s00415-026-14056-w (PMC13461926; doi:10.1007/s00415-026-14056-w)
Supplement: Supplementary file 1 [file 415_2026_14056_MOESM1_ESM.docx]

Supplementary Table 1: Comparison between included and excluded participants

| Variable | Included P. (final analysis) | Excluded P. (final analysis) | Missing in Excluded P.*1 |
| --- | --- | --- | --- |
| Number of participants | 894 | 185 | NA |
| Age (mean +/- SD) | 75+/-6 | 80+/-7 | 0 |
| Female (n/perc) | 439 (49) | 103 (56) | 0 |
| Men (n/perc) | 455 (51) | 82 (44) |  |
| ASR (mean+/-SD) | 2.21+/-0.8 | 2.46+/-1.15 | 74 |
| ASR (median/IQR) | 2/1 | 2/1 |  |
| TICS-m (mean+/-SD) | 35+/-5 | 33+/-6 | 74 |
| TICS-m (median/IQR) | 36/7 | 34/7 |  |
| Parkinson score (mean+/-SD) | 11+/-12 | 24+/-19 | 7 |
| Parkinson score (median/IQR) | 8/17 | 20/31 |  |
| Frail or prefrail (n,/perc) | 355/0.4 | 68/0.37 | 84 |
| Sleep (hours, mean+/-SD) | 7.7+/-1.4 | 8.8+/-2.9 | 6 |
| Sleep (hours, median/IQR) | 8/2 | 8/3 |  |
| Physical activity (PASE, mean+/-SD) | 121+/-55 | 91+/-60 | 54 |
| Physical activity (PASE, median/IQR) | 118/71 | 79/81 |  |
| Stroke (n/perc) | 63/7 | 32/17 | 0 |

‘1 the number of missing values in excluded participants is identical to the number of missing values in all participants because the final analysis was a complete cases analysis.

Supplementary Figure 1: Matrix of the co-occurrence of missing values. Diagonal (shaded): number of total missing values; Upper and lower triangles: number of co-occurent missing values

Supplementary Figure 2: Residual Mean Squared Error (RMSE) and adjusted R-squared during the variable selection. Dashed black line: Residual Mean Squared Error (RMSE)/20. Solid black line: adjusted R-squared during the variable selection process. Tremor is highlighted by red lines.
